# Heavy water recycling for producing deuterium compounds†

**DOI:** 10.1039/d2ra04369f

**Published:** 2022-08-31

**Authors:** Kazuhiro Akutsu-Suyama, Hironao Sajiki, Misaki Ueda, Makiko Asamoto, Yasuyuki Tsutsumi

**Affiliations:** Neutron Science and Technology Center, Comprehensive Research Organization for Science and Society (CROSS) Tokai Ibaraki 319-1106 Japan k_akutsu@corss.or.jp; Laboratory of Organic Chemistry, Gifu Pharmaceutical University Daigaku-nishi Gifu 501-1196 Japan; FC Development, Co., Ltd, N5, Ibaraki University 4-12-1 Nakanarusawacho Hitachi-shi Ibaraki 316-8511 Japan

## Abstract

Deuterium oxide (D_2_O) is a special variety of water that serves as a crucial resource in a range of applications, but it is a costly and unusual resource. We therefore developed a new D_2_O concentration system that combines a polymer electrolyte water electrolyzer and a catalytic combustor for recycling used D_2_O. In this study, 1.6 L of used D_2_O, with a concentration of 93.1%, was electrolyzed for 13.6 h to obtain 0.62 L of D_2_O, with a concentration of 99.3%. In addition, the recombined water obtained by burning electrolytic gas using the catalytic combustor was also electrolyzed for 8.8 h to obtain 0.22 L of D_2_O, with a concentration of 99.0%. The estimated separation factor of this electrolyzer at 25 °C was 3.6, which is very close to the equilibrium constant of the water/hydrogen isotope exchange reaction. Recycled D_2_O was used as a deuterium source for the deuteration reaction of sodium octanoate, and 93.6% deuterated sodium octanoate was obtained. It is concluded that there were no impurities in the recycled D_2_O that interfered with the deuteration reaction. These results can lead to the development of a cost-effective deuteration method for these materials.

## Abstract 2

Heavy water recycling for the production of deuterium compounds.

## Graphical abstract

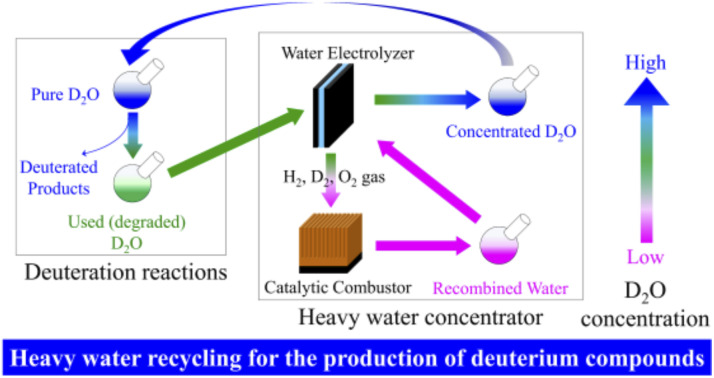

## Introduction

Deuterium-labeled compounds have been used to elucidate reaction mechanisms and/or kinetics, drug metabolism analysis, *etc.*^1–3^ Recently, deuterium-labeled compounds have been used not only in analytical reagents but also in novel industrial materials such as surface-deuterated silicon semiconductors^4,5^ and deuterated organic electro-luminescence elements.^6,7^ In addition, since the Food and Drug Administration recently approved deutetrabenazine (Austedo®) as a deuterated medicine,^8^ there has been a growing interest in deuterium-labeled compounds among pharmaceutical companies. In neutron studies, partially or fully deuterated compounds have been used to control the neutron scattering contrast of organic molecules and/or reduce the neutron incoherent scattering background of hydrogen.^9,10^

**Fig. 1:**
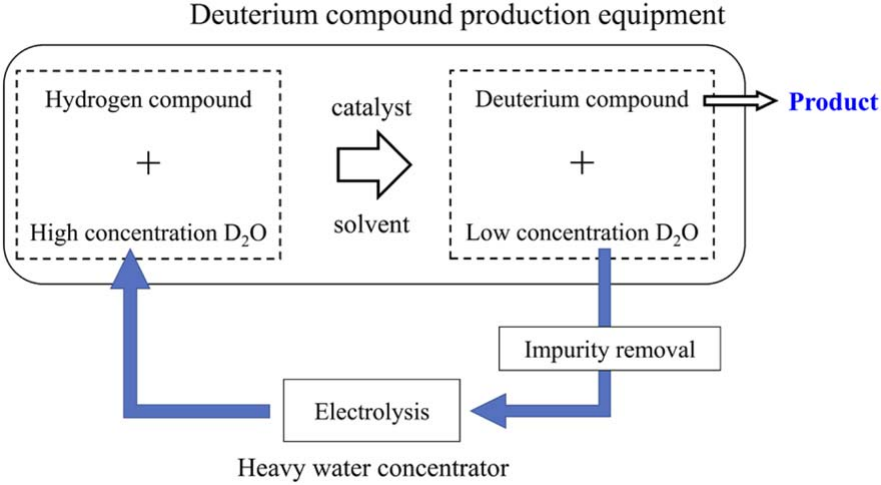
Heavy water recycling for the production of deuterium compounds.

Deuterium labeling reactions are usually carried out using deuterium oxide (D_2_O) as a deuterium source in the presence of protonated organic molecules and carbon-supported platinum-group metals, which serve as a catalyst.^9–18^ When the deuteration reaction has proceeded sufficiently, the concentration of deuterium in D_2_O decreases with increasing deuteration level of organic molecules. Recently, a deuterium labeling reaction using heavy water as a deuterium source and using a continuous flow reactor has also been reported.^19,20^ Since efficiently recycling used D_2_O from the reaction mixture is extremely difficult, the used D_2_O is disposed as industrial waste. However, depending on the amount of D_2_O used, that is, the demand for deuterium-labeled compounds, the recycling method of used D_2_O shown in Fig. 1 can have significant economic benefits.

This study aims to develop a new D_2_O recycling system for the production of deuterium compounds and to investigate the problems in its practical use. High purity D_2_O was first produced by electrolysis in 1933. In the 1960s, many studies were conducted on hydrogen isotope exchange reactions on electrodes.^21–24^ However, research on hydrogen isotope exchange reactions with polymer electrolyte water electrolyzers (PEWEs) capable of high current density operation has only recently begun.^25–28^

The D_2_O production method used in the Manhattan project was published in 1954, and since then many studies on industrial production methods of D_2_O have been reported.^29^ There are many methods for extracting high concentrations of D_2_O from natural water, which contains approximately 140 ppm of D_2_O, such as electrolysis, distillation, deep cold separation, water–hydrogen sulfide, and hydrogen–ammonia exchange reaction.

The electrolysis approach is inefficient since it uses a lot of energy even if isotope separation is easy.^30^ On the other hand, the energy consumption for electrolysis becomes small for recycling used D_2_O because the hydrogen concentration level is also small in the used D_2_O. Therefore, electrolysis is not always uneconomical. D_2_O recycling using the electrolytic method has been carried out for nuclear power and fusion.^31–33^Table 1 contrasts the existing Fugen D_2_O purification equipment with the new equipment that is being developed. In the D_2_O purification equipment for the Fugen nuclear power station,^31,32^ an alkaline electrolytic bath without a partition membrane was used as the water electrolyzer. The generated electrolytic gas was diluted with helium gas and recombined using a catalytic combustor to recover deuterium in the electrolytic gas as dilute D_2_O. In addition to the electrolyzer and combustor, a helium gas circulation system, a distiller for removing electrolytic aids in the electrolytic solution, and radiation control equipment were used. On the other hand, a new D_2_O concentrator to be developed handles a smaller amount of D_2_O than that used in the nuclear power and fusion fields. It does not handle radioactive substances; hence, it is required to be small, simple, and inexpensive rather than highly efficient.

**Table 1:** Comparison between conventional equipment and new equipment to be developed

Name	Conventional equipment	New equipment to be developed
	Fugen D_2_O purification equipment	2 L per day D_2_O concentrator
Intended use	Purification of D_2_O used as a moderator in nuclear reactors	D_2_O recycling for producing deuterium compounds

Electrolyser	Non-diaphragm alkaline electrolytic cell, current density 0.1 A cm^−2^, separation factor 10.5	PEM type electrolytic cell. Current density 2 A cm^−2^

Recombiner	Catalytic combustor with helium gas circulation system	PEFC or heat sink type catalytic combustor without gas circulation system

Treated D_2_O	15 L per day. Standard D_2_O concentration 95%	2 L per day. Standard D_2_O concentration 95%

Equipment size	14 m × 20 m × 10 m	Standard rack storage size

The new D_2_O concentrator uses a PEM (polymer electrolyte membrane)-type electrolysis cell that can operate at high current densities, making the electrolyzer much smaller and less expensive. The new D_2_O concentrator uses a PEFC (polymer electrolyte fuel cell) or heat sink type catalytic combustor as a recombiner, omitting the helium gas circulation system and greatly simplifying and miniaturizing the equipment.

In this study, the concentration characteristics of the concentrator were compared with the calculated values based on the law of conservation of substances and discussed quantitatively. In addition, deuteration experiments were carried out to check the quality of the recycled D_2_O obtained using the D_2_O concentrator. As fatty acids are one of the most interesting research areas for materials and life science researchers, developing a cost-effective deuteration method for these materials is an important step for the future application of deuterium labeling technologies. In this study, sodium octanoate was deuterated as the target material.

## Experimental

### Materials

Unless otherwise noted, materials obtained from commercial suppliers were used without further purification. Twice-distilled water was used in all the experiments. IrO_2_ catalyst (IrO_2_ black, 100%, 1.0 mg cm^−2^, Umicore AG & Co. KG), Pt/C catalyst (Pt 50 wt%, Pt 0.5 mg cm^−2^, NE Chemcat Co), Nafion NR212 and N117 membranes (50 μm and 180 μm thick, DuPont Inc), Ti porous substrate (200 μm thick, Bekaert Inc), sodium octanoate (99.0%, TCI Chemical Co), 1,4-dioxane (99.0%, TCI Chemical Co), deuterium oxide (D_2_O, 99.9% D, Cambridge Isotope Laboratories Inc.), and Pt/C for deuteration (10.0%, NE Chemcat Co.). A PEFC, PEWE, and catalytic combustor were supplied by FC Development, Co., Ltd.

### Preliminary test equipment

**Fig. 2:**
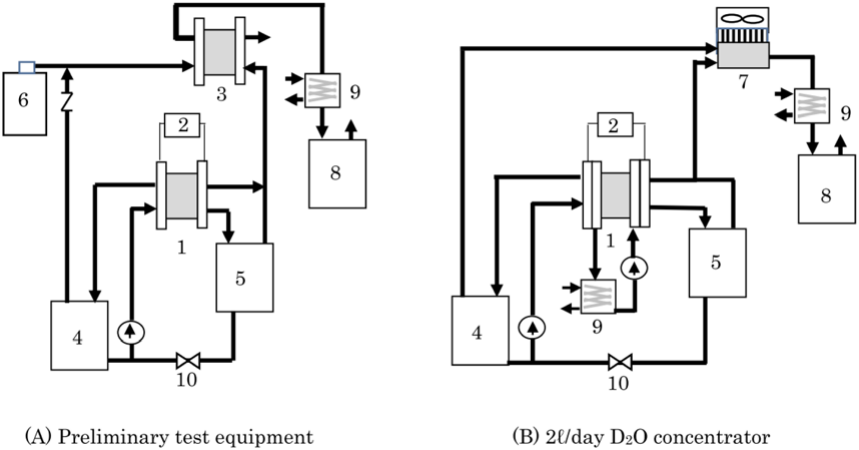
Schematic illustration of the heavy water concentrator of the preliminary test equipment (A) and the 2 L d^−1^ D_2_O concentrator (B). (1) PEWE, (2) power supply, (3) PEFC, (4) anode water tank, (5) cathode water tank, (6) air cylinder, (7) catalytic combustor, (8) recombined water tank, (9) chiller, (10) valve.

As a recombiner for 2 L per day concentrator, two types of equipment, PEFC and heat sink type catalytic combustor, were examined. Preliminary test equipment means equipment that uses PEFC as a recombiner. Fig. 2(A) shows a schematic diagram of a D_2_O concentrator that combines a PEWE and PEFC used in the preliminary test. Because high isotope separation efficiency has been reportedly expected by a method called combined electrolysis fuel cell (CEFC),^34–37^ it was investigated using the equipment in Fig. 2(A) to determine whether the method is suitable for the D_2_O recycling concentrator. For D_2_O concentration, there are volume reduction electrolysis methods or constant volume electrolysis methods.^29^ This concentrator was also used to validate the D_2_O concentration method.

The PEWE 1 used in this concentrator was a single cell with an electrode area of 4.18 cm^2^ or a 3 cell stack that uses single cells with the same electrode area. In this PEWE, anode catalysts of IrO_2_ (1 mg cm^−2^), cathode catalysts of Pt/C (0.5 mg cm^−2^), an electrolyte membrane of N117, a Pt-plated Ti porous anode gas diffusion layer, a water-repellent carbon paper cathode gas diffusion layer, and Pt-plated Ti flow plates were used. The PEFC 3 used in this concentrator was a single cell with an electrode area of 25 cm^2^. The amount of Pt load on each electrode was 0.5 mg cm^−2^, and an electrolyte membrane of NR212 was used.

Instead of taking used D_2_O, D_2_O obtained by diluting 99% D_2_O with ion-exchanged water was used in the preliminary test. Raw D_2_O (100 g) was put in anode water tank 4 and circulated to the PEWE 1 anode at a flow rate of 5 mL min^−1^ for a single cell and 15 mL min^−1^ for a 3 cell stack. The oxygen generated at the anode of PEWE 1 was supplied to the cathode of PEFC 3 together with air (92 mL min^−1^ in a single-cell test and 276 mL min^−1^ in a 3 cell stack test) from the cylinder. The cathode exhaust gas was cooled by chiller 9, and the recombined water was collected in the recombined water tank 8.

No gas or liquid was supplied to the cathode of PEWE 1, and the hydrogen generated at the cathode was supplied to the anode of PEFC 3, and the exhaust gas from the anode outlet was released to the atmosphere. The water that passed through the electrolyte membrane from the PEWE anode to the cathode was stored in the cathode water tank 5. The operating current of the PEWE was constant at 8.36 A (2 A cm^−2^), and the cell temperature was stable at 35 °C for a single cell and 42 °C for a 3 cell stack. Electrolysis was continued, and when anode water tank 4 started emptying, the lower valve 10 was opened, and the water accumulated in the cathode water tank 5 was returned to the anode water tank 4. Electrolysis was continued until the target time. Immediately after returning the cathode water to the anode tank, 5 mL of D_2_O in the anode tank was sampled, and its deuterium concentration was analyzed using an FT-IR spectrometer.

Because the PEFC must keep the electrolyte membrane wet, it is usually operated by supplying a humidified gas. It is desirable to humidify the recombined water in tank 8 so that the concentration of D_2_O does not change because of the mixing of humidified water. However, there was no recombined water at the start of the operation. Therefore, in this study, the non-humidifying operation lowered the cell temperature and suppressed the release of water vapor. An electronic load device was connected to the PEFC, and a constant current operation of 5 A (0.2 A cm^−2^) was performed for the PEWE single cell and 10 A (0.4 A cm^−2^) for the PEWE 3 cell stack. The hydrogen and oxygen utilization rates were 60% and 36% for the PEFC in the single-cell test, and 40% and 24% for the PEFC in the 3 cell stack test, respectively.

### 2 L per day D_2_O concentrator using heat sink type catalytic combustor for recombiner

Fig. 2(B) shows a schematic diagram of a 2 L per day D_2_O concentrator in which PEWE 1 and catalytic combustor 7 are combined. This is a small, simple, and inexpensive concentrator newly developed for D_2_O recycling and used to produce deuterium compounds. Here, 2 L per day means the ability to concentrate 2 L of 95% D_2_O to 99% within 24 h.

Using PEWE 1 with a separation membrane instead of an alkaline electrolytic bath without a separation membrane, the generated oxygen and hydrogen were separated, and helium dilution was not necessary. Hydrogen and oxygen generated by electrolysis are directly supplied to the catalytic combustor without dilution and burned, simplifying the concentrator. Unlike the alkaline electrolytic bath, the PEWE does not require the addition of an electrolyte; therefore, a distiller for removing the added electrolyte is not necessary.

Further simplification of the concentrator is possible by using only PEWE and excluding the catalytic combustor. However, it is necessary to re-electrolyze the recombined water to increase the recovery rate of the high-concentration D_2_O; therefore, the concentrator was equipped with a catalytic combustor.

Fig. 2(B) shows a concentrator that performs batch operations. Multi-stage electrolysis is required for continuous operation, which is extremely complicated^38^ and unsuitable for inexpensive concentrators. To improve the separation efficiency, a method called combined electrolysis catalytic exchange (CECE) that recovers deuterium in electrolyzed hydrogen gas with raw water is also reported.^34^ However, in a small-scale concentrator, it is desirable to simplify the system and reduce the initial cost of the concentrator rather than increasing the separation efficiency to shorten the operation time and reducing the running cost.

**Fig. 3:**
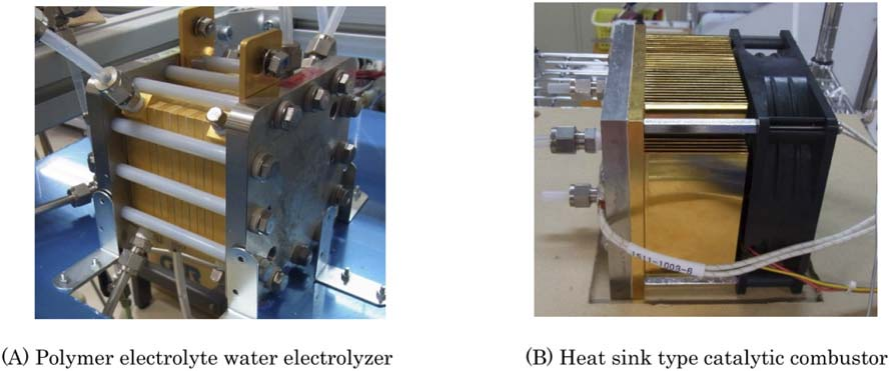
Photograph of the main components of the 2 L per day heavy water concentrator. (A) and (B) are the PEWE and the heat sink type catalytic combustor, respectively.

We chose the concentrator shown in Fig. 2(B), which consisted of an electrolyzer and a catalytic combustor. PEWE 1 used in this concentrator for the reasons mentioned above, in which four cells with an electrode area of 25 cm^2^ were stacked, and the external photograph is shown in Fig. 3(A). The materials for the electrodes, electrolyte membrane, flow plate, *etc.*, used for this stack are the same as those used for the PEWE in the preliminary test. The cooling water was circulated, and the cell temperature was kept constant at 25 °C.

A newly developed heat sink-type catalytic combustor was used as the catalytic combustor 7. In this combustor, a substrate carrying a combustion catalyst with an area of 67 cm^2^ was mounted onto the hot plate of the heat sink. The heat of combustion was released into the atmosphere *via* heat radiation fins attached to the hot plate. The fan was controlled to maintain a constant combustion temperature of 120 °C. An external photograph of the catalytic combustor is shown in Fig. 3(B).

Using this concentrator, a demonstration test of D_2_O recycling was conducted, in which D_2_O with a reduced concentration used in the production of deuterium compounds was concentrated and reused. Used D_2_O (1.61 L) was purified by distillation under reduced pressure, and it was placed in an anode water tank, circulated at 50 mL min^−1^, and concentrated by electrolysis at 50 A (2 A cm^−2^). Valve 10 was automatically opened and closed to return the water in cathode tank 5 to anode tank 4.

### Purification of used D_2_O

Because the used D_2_O solution, which was used to deuterate sodium octanoate, may contain some impurities (*e.g.*, sodium octanoate, catalyst, and metal ions that were eluted from the stainless steel reactor), these impurities were removed from the used D_2_O solution before recycling.

For the separation of these molecules from D_2_O, distillation is the best and most commonly used separation method. Therefore, a 500 mL round-bottom flask connected to a glass Liebig condenser was used with cooling water at 10 °C to purify the used D_2_O.

### Analysis of H and D concentrations

Because the estimation of the ratio of the two isotopes, that is, H and D atoms in used and recycled D_2_O is crucial for estimating the efficiency of the D_2_O recycling system, infrared (IR) spectroscopy was used to analyze H and D concentrations in the presence of H_2_O and D_2_O. IR spectroscopy is a general method used to estimate H and D concentrations.^39^

**Fig. 4:**
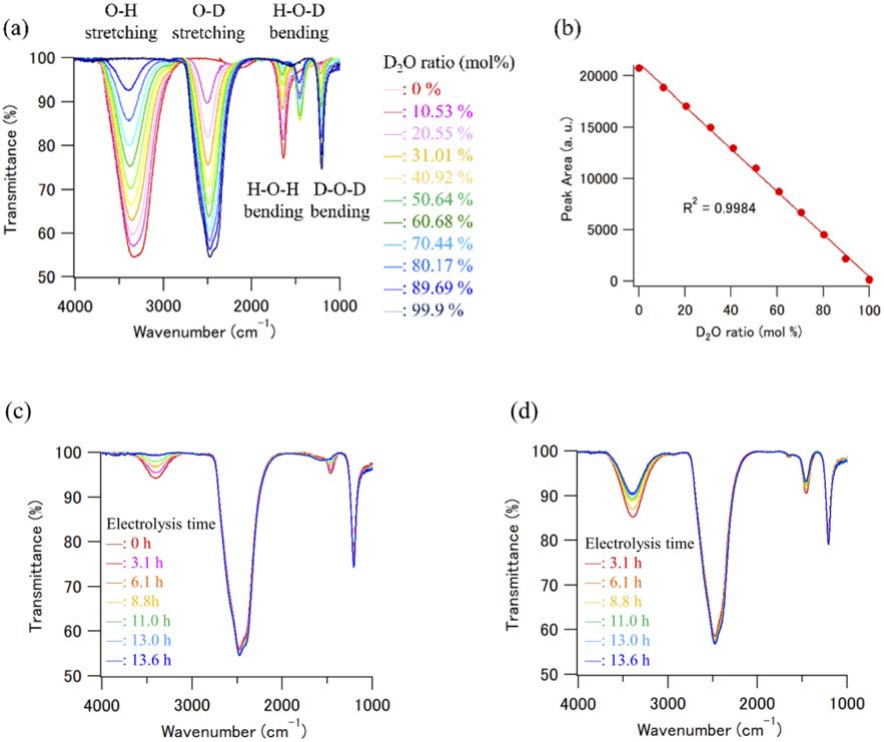
(a) The calibration data set of H_2_O–D_2_O mixtures obtained by FT-IR ATR. (b) The plot of the peak area of O–H stretching region *vs.* the initial concentration ratio of D_2_O/H_2_O mixture. The error bars are small enough to be within the symbols. (c) The FT-IR ATR spectra of the D_2_O and D_2_O concentrated samples. (d) The FT-IR ATR spectra of recombined D_2_O samples.

Fourier transform infrared (FT-IR) spectra were measured using an FT/IR-4100ST (Nihon Bunko Co. Ltd, Tokyo, Japan) system equipped with an attenuated total reflectance (ATR) unit (PRO670H-S, Nihon Bunko Co. Ltd, Tokyo, Japan). The wavenumber range and resolution were 700–4000 and 4 cm^−1^, respectively. Each spectrum was determined from an average of four scans, and all measurements were performed at ambient temperature (20 °C). Fifteen microliters of D_2_O solution were placed on the ZnSe crystal of the ATR unit for measurements, and all FT-IR spectra measurements were completed within 30 s, because a certain degree of H_2_O contamination from the air clearly occurred after 60 s on the ZnSe crystal. The concentration of D in the D_2_O sample solutions was determined from a calibration curve prepared using the peak area of the O–H stretching region. Fig. 4(a) shows the calibration data set of the H_2_O–D_2_O mixtures obtained by FT-IR ATR. The intensity of the O–H stretching absorption peak decreased with increasing D_2_O concentration in the D_2_O/H_2_O mixture. The plot of the peak area of the O–H stretching region *vs.* the initial concentration ratio of the D_2_O/H_2_O mixture, which was determined by the mixing weight of the D_2_O/H_2_O mixture, was well matched (*R*^2^ = 0.9984, Fig. 4(b)). We prepared some calibration curves which were obtained by plotting the integral of the peak at the O–H stretching band and the O–D stretching band (Fig. S1†). As a result, we concluded that the calibration curve obtained by plotting the integral of the peak at the O–H stretching band is the best to calculate the deuterium concentration of the D_2_O samples. Therefore, the calibration curve obtained using the linear regression method was used to estimate the D_2_O concentration of the recycled D_2_O samples (Fig. 4(c) and (d)). Note that the obtained standard deviation for three consecutive experiments was 0.068 (Fig. S2 and Table S1†). Therefore, it can be said that this analytical method has high reproducibility for the purity analysis of D_2_O samples.

### Direct deuteration of sodium octanoate

Deuteration reactions were carried out in a stainless steel reactor (TSSR, TPR1-VSI-300, SUS316, Taiatsu Techno Corp., Tokyo, Japan).^40^

A mixture of sodium octanoate-*h*_15_ (5.0 g, 30.1 mmol) and Pt/C (10% Pt, 0.65 g, 0.33 mmol) in new or recycled D_2_O (100 mL) was loaded into the TSSR. The mixture was vacuum degassed for 10 min to remove oxygen. The reactor was purged with H_2_ for 10 s and then sealed. The mixture was heated at 220 °C in an oil bath and stirred continuously for 72 h. After cooling to 20 °C, the contents were filtered through a short plug of Celite to remove the catalyst and washed a second time with H_2_O (50 mL). The filtrate was evaporated to dryness under reduced pressure. The yields of deuterated sodium octanoate using the new and recycled D_2_O solutions were 85.9% and 90.7%, respectively. The deuteration ratio was determined by ^1^H NMR spectroscopy using D_2_O, with 1,4-dioxane as an internal standard, and ^2^H NMR spectroscopy using H_2_O. The ^1^H and ^2^H spectra were recorded on a BRUKER AVANCE III 400 spectrometer and a JEOL JMTC-400/54/JJ/YH spectrometer (^1^H: 400 MHz, ^2^H: 61.4 MHz).

## Results and discussion

### Preliminary test – PEWE and PEFC cell voltages

**Fig. 5:**
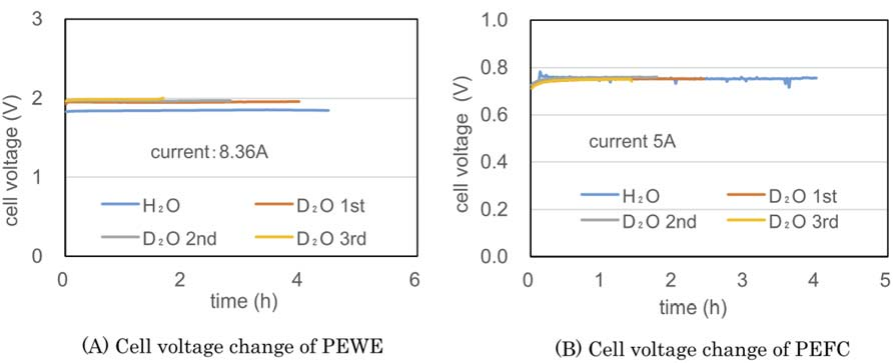
The cell voltages of PEWE (A) and PEFC (B) during the combined operation of a PEWE single cell at 8.36 A and PEFC at 5 A using the preliminary test equipment. The blue lines are the test result obtained using ion-exchanged water. The red, gray, and yellow lines result from the first, second, and third operations using D_2_O, respectively.

Fig. 5(A) and (B) show the cell voltages of PEWE and PEFC during the combined operation of a PEWE single cell at 8.36 A and PEFC at 5 A using the preliminary test equipment, respectively. The H_2_O line in Fig. 5(A) was obtained using ion-exchanged water before the D_2_O test. In the H_2_O electrolysis characteristics of Fig. 5(A), the cell voltage is 1.8 V at 2 A cm^−2^, indicating PEWE with normal characteristics. The PEFC in Fig. 5(B) also shows a normal characteristic of 0.75 V at 0.2 A cm^−2^.

The initial concentration of D_2_O used in the test was 95%. The D_2_O 1st line in Fig. 5(A) was the test result obtained until the amount of D_2_O initially put into the anode was almost empty. The D_2_O 2nd and 3rd lines represent the second and third operations, respectively, after moving water from the cathode water tank to the anode water tank. Because the amount of water decreased during electrolysis, the electrolysis time was shortened from 4.0 h for the first cycle to 2.8 h for the second cycle and 1.7 h for the third cycle. The cell voltage of H_2_O was kept constant at 1.9 V, and the cell voltage of D_2_O was kept constant at 2 V with almost the same data for 1st, 2nd, and 3rd operations. D_2_O is more difficult to electrolyze than H_2_O with 0.1 V.

The cell voltage of PEFC shown in Fig. 5(B) was approximately 0.76 V, but a pulse-like cell voltage drop was occasionally observed. Despite the non-humidifying operation, the cell temperature was as low as 32–34 °C; hence, it is presumed that water droplets accumulated in the gas channel, and a pulsed cell voltage drop occurred.

The appearance of the pulse-shaped voltage drop suggests that stable operation will be difficult if the current is increased any further. Fig. 5 recombines the gas generated by electrolysis at 8.36 A at 5 A. Hydrogen utilization rate is very low. Since hydrogen containing a large amount of deuterium is discarded, it is in an unfavorable state for a heavy water concentrator.

### Preliminary test – D_2_O concentration characteristics

**Fig. 6:**
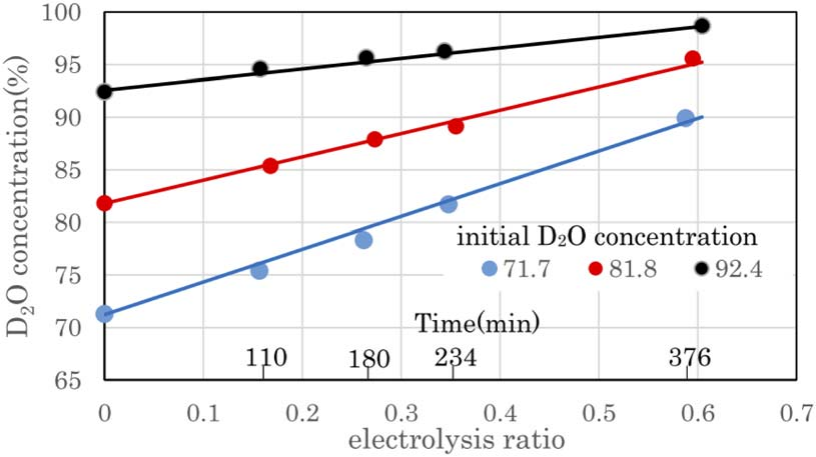
D_2_O concentration change by electrolysis using the preliminary test equipment. D_2_O concentration values determine the filled circles, and solid lines are calculated using the volume reduction electrolysis formula. The error bars are small enough to be within the symbols.

Fig. 6 shows the change in D_2_O concentration in the anode water tank during combined operation with a 8.36 A 3 cell stack PEWE and a PEFC single-cell 10 A in the preliminary test equipment. D_2_O with concentrations of 71.7%, 81.8%, and 92.4% were prepared by diluting commercial D_2_O (99.9%) with ion-exchanged water. The electrolysis ratio on the horizontal axis represents the ratio of electrolyzed water moles to raw water. The moles of raw water were calculated from the weight and D_2_O concentration of raw water. The moles of electrolyzed water were calculated from the current, the number of stacked cells in the stack, and the electrolysis time. When electrolyzed to an electrolysis ratio of approximately 0.6, the D_2_O concentration increased from 71.7% to 89.9%, 81.8% to 95.6%, and 92.4% to 98.7%, respectively.

The time on the horizontal axis shows the electrolysis time of D_2_O with an initial concentration of 71.7%. The first, second, third, and ninth times were 110 min, 180 min, 234 min, and 376 min, respectively, when the anode water tank was almost empty, and water was moved from the cathode water tank to the anode water tank. Because the amount of water decreased with electrolysis, the time pitch for moving water gradually decreased. Fig. 6 shows that the D_2_O concentration increased as water decreased owing to electrolysis. This concentration method is called the volume-reduction electrolysis method. The graph shown in black (●) in Fig. 6 is the measured value, and the solid line close to the straight line is the calculated value using the volume reduction electrolysis formula.^30^ The measured value of the initial D_2_O concentration and the separation coefficient of 2.9 was used in the calculation.

The separation coefficient *α* is defined by the following equation.^30^1
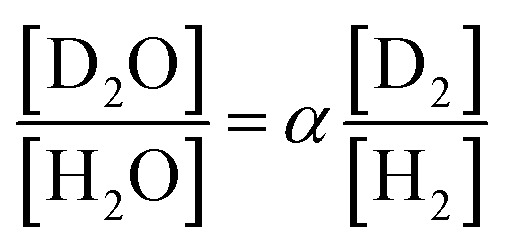


Here, [D_2_O] indicates the concentration of each component molecule. When there are no impurities other than D, the relationship between the D_2_O concentration *P* and the deuterium gas concentration *P*′ can be expressed by eqn (2).2
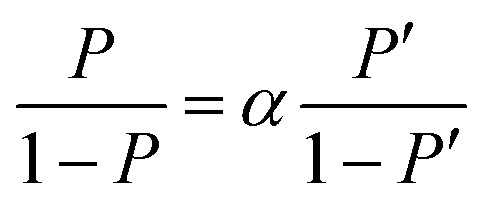


The formula for the volume reduction electrolysis is as follows.^30^3
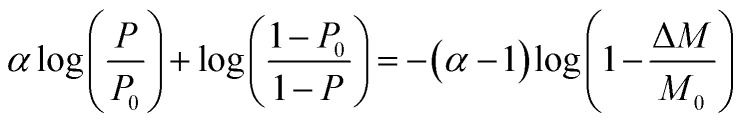


Here, *P*_0_ is the initial D_2_O concentration, and Δ*M*/*M*_0_ is the electrolysis ratio. *M*_0_ is the number of moles of initial water, and Δ*M* is the number of moles of electrolyzed water. This equation was derived based on the law of conservation of substances in the volume reduction electrolysis process for an electrolyzer without a membrane between the anode and cathode. Therefore, the calculated solid line in Fig. 6 is compared with the measured value in the state where the waters of the anode and cathode were mixed. An equation describing the concentration characteristics of an electrolyzer with a membrane that separates the anode and cathode has not yet been reported. Using eqn (3), the D_2_O concentration was calculated by changing the separation coefficient (*α*) and comparing it with the measured values. As a result, a good agreement was obtained, as shown in Fig. 6, when *α* = 2.9. Considering that the measured values of D_2_O concentration at 12 points excluding the initial D_2_O concentration are almost equal to the calculated values at only one value of *α* = 2.9 means that eqn (3) is useful for predicting electrolytic concentration characteristics.

As shown in Fig. 6, the D_2_O concentration increased continuously with electrolysis. However, the D_2_O concentrations of the cathode and anode waters during electrolysis were known to be almost constant,^25,27^ and they changed stepwise as water was transferred from the cathode tank to the anode tank.

### Preliminary test – recombination of electrolytic gas by PEFC

To adopt PEFC as an electrolytic gas recombination device for D_2_O recycling, the following issues must be resolved.

#### (1) Improvement of air line

PEFCs are difficult to operate with a high oxygen utilization rate exceeding 60%. Therefore, the dry air of cylinder 6 was added, as shown in Fig. 2(A). D_2_O recycling concentrators produced high concentrations of D_2_O. When using air from the atmosphere, it is necessary to dry the air to an extremely low dew point to prevent a decrease in the D_2_O concentration.

#### (2) Hydrogen utilization

In D_2_O recycling, high-concentration D_2_O is electrolyzed, so the concentration of deuterium in the electrolytic gas is also high. A low hydrogen utilization rate leads to a decrease in the recovery rate of recombined water. The hydrogen utilization rate must be 90% or higher. However, as mentioned above, it is difficult to stably operate a PEFC at a high hydrogen utilization rate.

#### (3) Non-humidifying operation

At startup, insufficient concentration of D_2_O is available for humidification. PEFCs capable of operating under non-humidifying conditions are required.

The CEFC method is not suitable for D_2_O recycling, which requires high concentrations of D_2_O. Therefore, in the 2 L per day D_2_O concentrator, we have developed a new system that uses a catalytic combustor that directly burns the electrolytic gas without mixing other gases instead of PEFC. Moreover, it should be noted that this equipment has already repeated the heavy water concentration cycle for 350 hours and continues to operate stably.

### D_2_O concentration characteristics using 2 L per day D_2_O concentrator

**Fig. 7:**
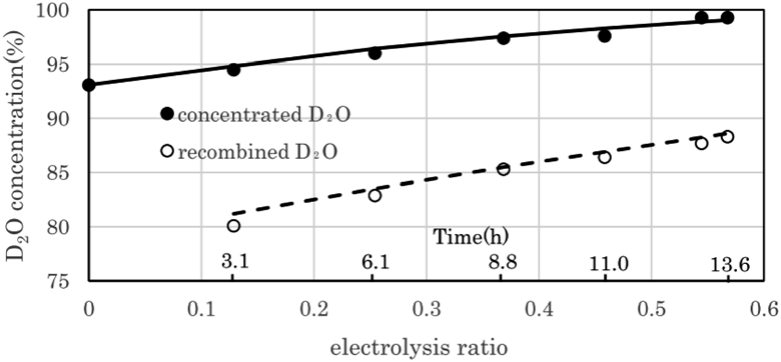
Performance of 2 L per day D_2_O concentrator. The filled circles represent experimentally determined D_2_O concentration values obtained using the 2 L per day D_2_O concentrator. The solid line represents the D_2_O concentration calculated using the separation coefficient *α* (3.6). The open circles represent experimentally determined D_2_O concentration values of the recombined water in the tank, and the dotted line represent calculated recombined D_2_O concentration. The error bars are small enough to be within the symbols.

Fig. 7 shows the change in D_2_O concentration in the test in which 1.6 L of used D_2_O with an initial concentration of 93.1% was concentrated using the 2 L per day D_2_O concentrator. ● is the concentration in the anode water tank for the PEWE, and ○ is the D_2_O concentration in the recombined water tank for the catalytic combustor.

The solid line is the D_2_O concentration (*P*) calculated by substituting the measured initial concentration (*P*_0_) (93.1%) and the separation coefficient (*α* = 3.6) into the volume reduction electrolysis formula in eqn (3). When the separation coefficient was 3.6, the measured values were almost on the calculated curve, as shown in Fig. 7. The dotted line represents the calculated recombined water concentration obtained, assuming that the initial D_2_O is divided into concentrated and recombined water. The measured concentration of the recombined water (○) in Fig. 7 was almost on the calculated dotted line. This is a reasonable result because it is based on the law of conservation of substances. The separation coefficient of 3.6 in Fig. 7 was significantly larger than the separation coefficient of 2.9 in Fig. 6. One of the reasons for this difference was the difference in operating temperature. The PEWE cell temperature in Fig. 6 was 42 °C, whereas that in Fig. 7 was set to 25 °C. It is well known that the separation coefficient increases as the operating temperature decreases.^22,41,42^ The equilibrium constant (K) of the isotope exchange reaction of the following equation on the catalyst was 3.62 at 25 °C and 3.20 at 50 °C.^30^

4 H_2_O(vap) + HD(gas) ⇄ HDO(vap) + H_2_(gas)

5
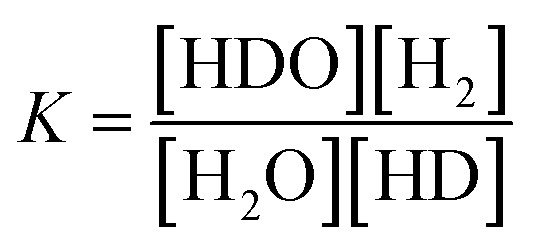


The separation coefficient of 3.6 in Fig. 7 is close to the equilibrium constant of 3.62 in eqn (4). However, the separation coefficient of 2.9 (in Fig. 6) is smaller than the equilibrium constant of 3.2 at 50 °C.

As the amount of raw water used for the experiment (in Fig. 6) is as small as 100 g, it may be owing to the influence of H_2_O contamination from the atmosphere.

### Recombination of electrolytic gas using a heat sink type catalytic combustor

**Fig. 8:**
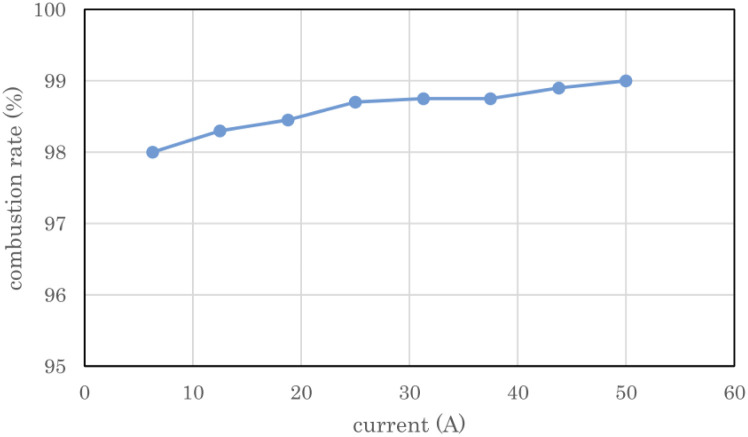
Combustion rate of heat sink-type catalyst combustor for 2 L per day heavy water concentrator. The error bars are small enough to be within the symbols.

Fig. 8 shows the measured combustion rate of the heat sink-type catalytic combustor used in the 2 L per day D_2_O concentrator. The flow rate of the electrolytic gas supplied to the catalytic combustor was changed by changing the PEWE current. The combustion rate on the vertical axis represents the ratio of the burned gas to the supplied gas. The exhaust gas from combustor 7 was dehumidified through chiller 9 using the concentrator shown in Fig. 2(B), and the exhaust gas flow rate of the recombined water tank 8 was measured and used as the flow rate of the unburned gas. As shown in Fig. 8, at a rated current of 50 A for the PEWE, the combustion rate of the combustor was 99%, which was high enough for practical use.

### Re-electrolysis of recombined water

To increase the recovery rate of D_2_O, 88.3% of the recombined water recovered in the test (shown in Fig. 7) was further electrolyzed to a D_2_O concentration of 99% or higher. The measured results are presented in Table 2.

**Table 2:** Recovery of concentrated D_2_O from used D_2_O^a^

Electrolysis condition	Sample name	*C* (%)	Sample volume (*L*)	
			Measured	Theoretical
Initial	Used D_2_O	93.1	1.61	—

After 13.6 h electrolysis of used D_2_O	Concentrated D_2_O-1	99.3	0.62	0.70
	Recombined D_2_O-1	88.3	0.86	0.91

After 8.8 h electrolysis of recombined D_2_O-1	Concentrated D_2_O-2	99.0	0.22	0.27
	Recombined D_2_O-2	83.6	0.56	0.59

a *C*: deuterium concentration of the sample.

In the first 13.6 h of electrolysis, 1.61 L of 93.1% used D_2_O was separated into 0.62 L of 99.3% concentrated D_2_O-1 and 0.86 L of 88.3% recombined D_2_O-1. The recovery rate of D_2_O with a concentration of 99% or more with respect to that of the used D_2_O was 38.5%. When the recombined water D_2_O-1 was electrolyzed for 8.8 h, 0.22 L of 99% concentrated D_2_O-2 was obtained, and the total amount of D_2_O with a concentration of 99% or more at a total electrolysis time of 22.4 h was 0.84 L, and the recovery rate relative to the used D2O increased to 52.2%. From 1.61 L of used D_2_O, a total of 1.4 L of concentrated D_2_O-1 (0.62 L), concentrated D_2_O-2 (0.22 L), and recombined D_2_O-2 (0.56 L) was obtained, and 0.21 L was missing. The theoretical column in Table 2 lists the calculated values of the electrochemical equivalents. The measured volumes of concentrated D_2_O-1 and concentrated D_2_O-2 were 88.6% and 81.5% of the theoretical volume, respectively. The main reason for such low ratios is the sampling loss of D_2_O in concentration measurements. However, the measured theoretical ratios of the recombined D_2_O-1 and recombined D_2_O-2 were 94.5% and 94.9%, respectively, which were lower than the 99% predicted from the measured exhaust gas flow rate in Fig. 8. It is obvious that these values were sufficiently high for practical use.

### Evaluation of new D_2_O concentrator

#### (1) D_2_O recovery

Using the D_2_O concentrator, 0.84 L of D_2_O with a concentration of >99% was recovered from the 1.6 L of D_2_O with a concentration of 93.1% that was previously discarded. This method is very useful because the production of high-concentration heavy water from natural water is extremely difficult and consumes a large amount of energy.

#### (2) Energy consumption

The rated electrolytic power of the electrolyzer is 500 W. The power for cooling of this electrolyzer and recombiner is 1.3 kW and 400 W, respectively. Since the total power of the concentrator is 2.2 kW, it consumes 49.3 kW h of energy in 22.4 hours of operation. Therefore, the electricity cost required for the tests in Table 1 was around $11; see the ESI† details. Considering that D_2_O costs around $1000 per liter, electricity costs are negligibly low.

#### (3) Equipment durability

A key factor affecting the economics of this system is the durability of the equipment. The deterioration rate of the PEM type water electrolysis cell is 0.2 mV or less at 1000 h,^44,45^ and durability of 10 000 h or more is expected. There is also a report demonstrating 40 000 h operation.^46^ On the other hand, there are no reports on the durability of D_2_O concentrators. This equipment has already repeated the D_2_O concentration cycle for 350 hours and continues to operate stably. It is necessary to continue to operate this equipment and confirm its durability.

### Deuteration of sodium octanoate using new and recycled D_2_O

To check the quality of recycled D_2_O, comparative deuteration experiments were carried out using new (99.9%) and recycled (99.3%) D_2_O.

Fig. S2† shows the ^1^H and ^2^H NMR spectra of deuterated sodium octanoates. The deuteration efficiency was evaluated using ^1^H and ^2^H NMR spectroscopy, and the respective spectra were recorded. The estimated mean deuteration levels of sodium octanoate obtained using the new and recycled D_2_O were 94.7 and 93.6%, respectively. Although recycled D_2_O was used as a deuterium source for the deuteration reaction, the deuteration efficiency did not change significantly. This indicated that the recycled D_2_O did not contain any deuteration reaction inhibitors, that is, we were able to utilize the recycled D_2_O in the deuteration reaction. In addition, because the mean deuteration level of saturated fatty acids using the direct deuteration method was 94–98%, the obtained deuteration levels are consistent with those reported in the literature (ref. 10 and 43). Therefore, it is clear that there is no significant difference between the new and recycled D_2_O in the overall deuteration efficiency of the reaction.

## Conclusions

Recently, various deuterium-labeled compounds have been developed for new applications, and their demand is increasing. High concentrations of D_2_O were used to produce them, and used D_2_O, whose concentration decreased during the production process, was discarded as industrial waste. The purpose of this study is to enable the reconcentration and reuse of this used D_2_O. A PEWE was used for concentration, and the formula for substance preservation was able to describe its concentration characteristics. The recombination of electrolyzed gas is required to increase the amount of D_2_O recovered. The method of using fuel cells for recombination is not suitable for producing high concentrations of D_2_O.

In this study, we developed a new simple D_2_O concentrator that combines a 4-cell stack PEWE with an electrode area of 25 cm^2^ and a heat sink-type catalytic combustor for recombination. The D_2_O recycling test was conducted for the production of deuterated sodium octanoate using this concentrator.

Used D_2_O (1.6 L) with a final concentration of 93.1% after removing impurities by distillation was electrolyzed for 13.6 h to obtain 0.62 L of D_2_O with a concentration of 99.3%. The estimated separation factor of this electrolyzer at 25 °C was 3.6, which is very close to the equilibrium constant of the water/hydrogen isotope exchange reaction. By further electrolyzing the recombined water obtained by burning electrolytic gas with a catalytic combustor for 8.8 h, 0.22 L of 99% D_2_O was obtained.

It was confirmed that the deuteration levels of sodium octanoate obtained using the new and used D_2_O were almost the same. There were no impurities in the recovered D_2_O that interfered with the deuteration reaction. With an increasing demand for deuterium compounds, the need for D_2_O recycling in the production process is expected to increase.

## Author contributions

Conceptualization, K. A.-S., H. S., and Y. T.; data curation, K. A.-S., M. U., and Y. T.; formal analysis, K. A.-S., M. U., and Y. T.; funding acquisition, Y. T.; investigation, K. A.-S., M. U., M. A., and Y. T.; methodology, K. A.-S., H. S., and Y. T.; project administration, K. A.-S., H. S., and Y. T.; resources, K. A.-S. and Y. T.; software, K. A.-S.; supervision, H. S. and Y. T.; validation, K. A.-S., M. A., and Y. T.; visualization, K. A.-S. and Y. T.; writing – original draft, K. A.-S. and Y. T.; writing – review & editing, K. A.-S., H. S., M. U., M. A., and Y. T. All authors have read and agreed to the published version of the manuscript.

## Conflicts of interest

The authors declare no competing financial interest.

## Supplementary Material

RA-012-D2RA04369F-s001
