# Supplementary material for: Heavy water recycling for producing deuterium compounds
Source: RSC Adv. 2022 Aug 31;12(38):24821–9. doi: 10.1039/d2ra04369f (PMC9430630; doi:10.1039/d2ra04369f)
Supplement: RA-012-D2RA04369F-s001 [file RA-012-D2RA04369F-s001.pdf]

Electronic Supplementary Material (ESI) for RSC Advances.
This journal is © The Royal Society of Chemistry 2022

Supporting Information

Heavy water recycling for producing deuterium compounds

Kazuhiro Akutsu-Suyama,*[a] Hironao Sajiki,[b] Misaki Ueda,[a] Makiko Asamoto,[c] and Yasuyuki Tsutsumi*[c]

[a] Neutron Science and Technology Center, Comprehensive Research Organization for Science and Society (CROSS), Tokai, Ibaraki 319-1106, Japan

[b] Laboratory of Organic Chemistry, Gifu Pharmaceutical University, Daigaku-nishi, Gifu 501-1196, Japan

[c] FC Development, Co., Ltd., N5, Ibaraki University, 4-12-1 Nakanarusawacho, Hitachi-shi, Ibaraki 316-8511, Japan

Table of Contents

1. Calibration curves in the FTIR analysis S3

2. Confirming the reproducibility of the FTIR measurements S2

3. ^{1}H NMR of deuterated sodium octanoate S4

4. Estimation of the electricity cost for the D_2O recycling test S6

5. References S6

* Corresponding authors: E-mail address: k_akutsu@cross.or.jp and tsutsumi@fcdevelopment.co.jp

S1. Confirming the reproducibility of the FTIR measurements

The calibration curves, which were obtained by plotting the integral of the peak at the O-H stretching band and the O-D stretching band, were prepared to find the best calibration curve. Additionally, a calibration curve, which was obtained by plotting the area ratio of the O-H stretching band/O-D stretching band, was also prepared. These results indicated that the calibration curve obtained by plotting the integral of the peak at the O-H stretching band is the best. Although the linearity of the calibration curve improves when the plotting range of the calibration curve is narrowed (green plots), similar deuterium concentration values can be obtained using either calibration curve.

(a) Calibration curves obtained by the O-H stretching band.

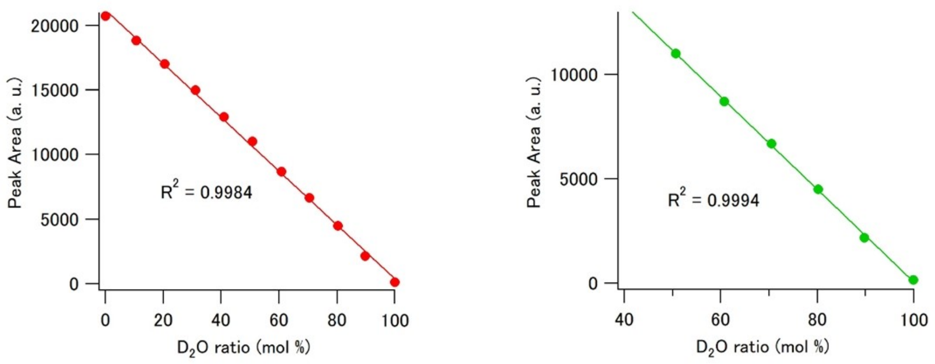

(b) Calibration curves obtained by the O-D stretching band.

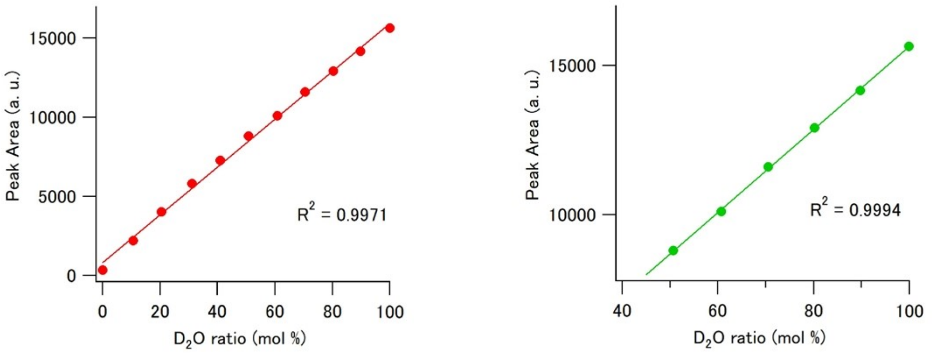

(c) Calibration curves obtained by the area ratio of the O-H stretching band/O-D stretching band.

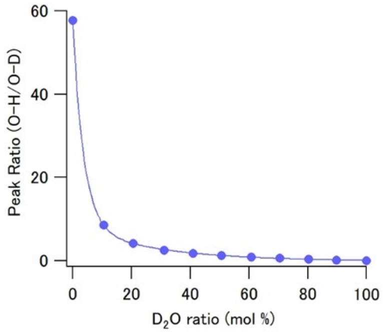

Figure S1 (a) The calibration curve obtained by plotting the integral of the peak at the O-H stretching band. (b) The calibration curve obtained by plotting the integral of the peak at the O-D stretching band. (c) The calibration curve obtained by plotting the area ratio of the O-H stretching band/O-D stretching band.

S2. Confirming the reproducibility of the FTIR measurements

Fourier transform infrared (FTIR) spectra were measured using an FT/IR-4100ST (Nihon Bunko Co. Ltd., Tokyo, Japan) system equipped with an attenuated total reflectance (ATR) unit (PRO670H-S, Nihon Bunko Co. Ltd., Tokyo, Japan). Repeat the measurement three times and take the average value and standard deviation (S.D.). The standard deviation was calculated by repeat the measurement three times.

Table S1. The concentration and standard deviation values of a 99.3% D_2O determined by the FTIR analysis.

	Purity	S.D.
Measurement 1	99.39 %	
Measurement 2	99.35 %	
Measurement 3	99.23 %	
Average	99.32 %	0.068

Figure S2. The FT-IR ATR spectra of the recycled D_2O (99.3 %) samples.

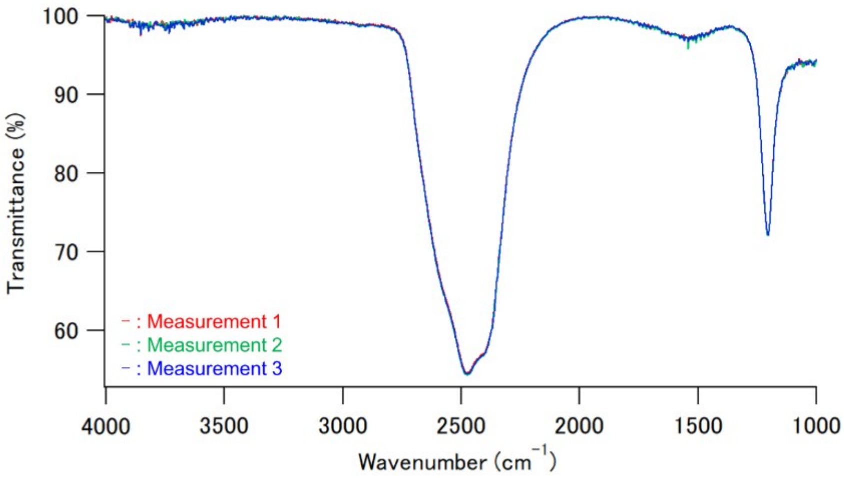

S3. ^1H and ^2H NMR spectra of deuterated sodium octanoates

The ^1H and ^2H NMR spectra of deuterated sodium octanoates were recorded using a 400 MHz NMR spectrometer (BRUKER AVANCE III 400 spectrometer and JEOL JMTC-400/54/JJ/YH spectrometer). The ^1H and ^2H NMR data are shown in below:

(A) ^1H NMR spectrum of the deuterated sodium octanoate synthesized using new D_2O

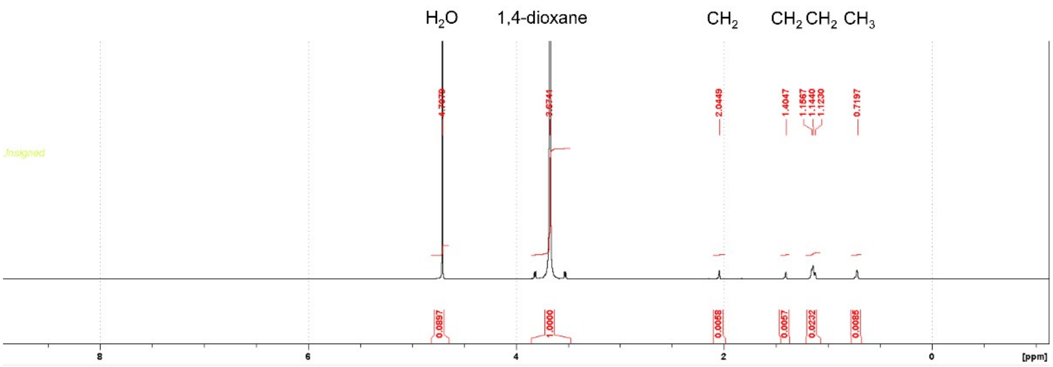

^2H NMR spectrum of the deuterated sodium octanoate synthesized using new D_2O

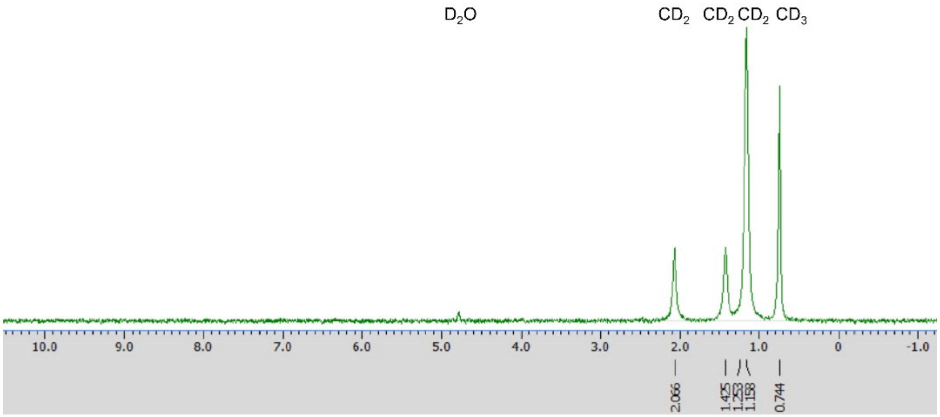

(B) ^{1}H NMR spectrum of the deuterated sodium octanoate synthesized using recycled D_2O

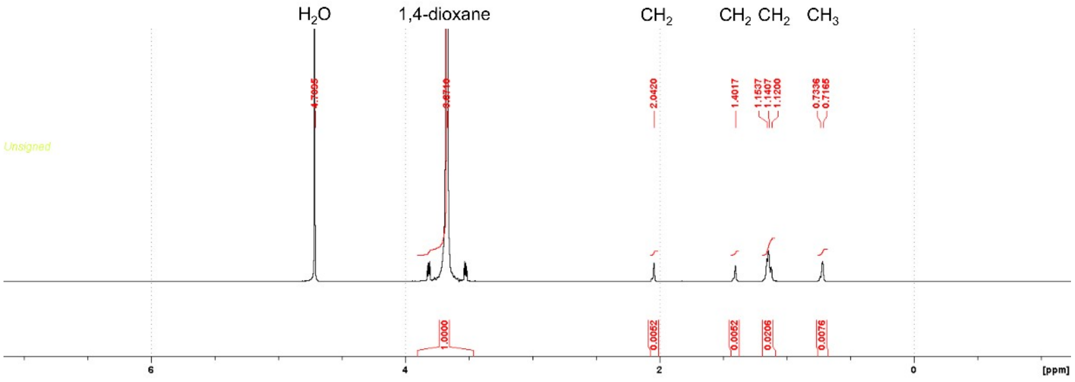

^{2}H NMR spectrum of the deuterated sodium octanoate synthesized using recycled D_2O

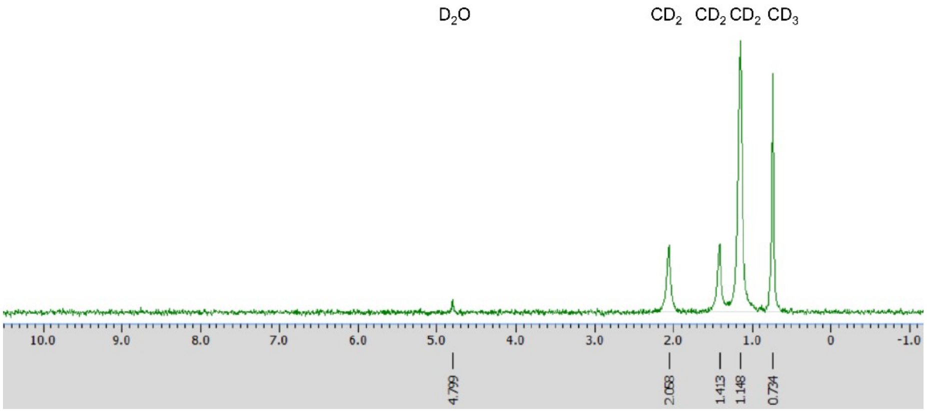

Figure S3. ^{1}H and ^{2}H NMR spectra of the deuterated sodium octanoate in D_2O or H_2O. (a) ^{1}H and ^{2}H NMR of the deuterated sodium octanoate synthesized using new D_2O (99.9%). ^{1}H NMR (400 MHz, D_2O, 1,4-dioxane) δ 0.72 (residual signal), 1.12–1.16 (residual signal), 1.40 (residual signal), 2.04 (residual signal). ^{2}H NMR (61.4 MHz, H_2O) δ 0.74 (br s), 1.16 (br s), 1.43 (br s), 2.07 (br s). (b) ^{1}H and ^{2}H NMR of the deuterated sodium octanoate synthesized using recycled D_2O (99.3%). ^{1}H NMR (400 MHz, D_2O, 1,4-dioxane) δ 0.71–0.73 (residual signal), 1.12–1.15 (residual signal), 1.40 (residual signal), 2.04 (residual signal). ^{2}H NMR (61.4 MHz, H_2O) δ 0.73 (br s), 1.15 (br s), 1.41 (br s), 2.06 (br s). 1,4-Dioxane: deuteration rate reference, D_2O and H_2O: NMR solvents.

S4. Estimation of the electricity cost for the D_2O recycling test

The electricity cost for the D_2O recycling test in this research was estimated with reference to the electricity rate of Tokyo Electric Power Company Holdings, Incorporated (TEPCO). The electricity rate for the Standard S Plan (TEPCO) is 30.57 yen per 1 kWh at the highest.[1] Since the total power of the concentrator is 2.2 kW, it consumes 49.3 kWh of energy in 22.4 hours of operation. Therefore, the electricity cost required for the D_2O recycling test was 1506 yen (ca. 11 US dollars).

References

1. Tokyo Electric Power Company Holdings Online, https://www.tepco.co.jp/ep/private/plan/standard/kanto/index-j.html (accessed August 2022).
